# *Quo Vadis* Clozapine? A Bibliometric Study of 45 Years of Research in International Context

**DOI:** 10.3390/ijms160923012

**Published:** 2015-09-23

**Authors:** Francisco López-Muñoz, Javier Sanz-Fuentenebro, Gabriel Rubio, Pilar García-García, Cecilio Álamo

**Affiliations:** 1 Faculty of Health Sciences and Chair of Genomic Medicine, Camilo José Cela University, C/Castillo de Alarcón, 49, Urb. Villafranca del Castillo, 28692 Villanueva de la Cañada, Madrid, Spain; 2 Department of Biomedical Sciences (Pharmacology Area), Faculty of Medicine and Health Sciences, University of Alcalá, Campus Universitario-C/19, Ctra. Madrid-Barcelona, Km. 33,600, 28871 Alcalá de Henares, Madrid, Spain; E-Mails: pigarcia@grupojuste.com (P.G.-G.); cecilioalamo@hotmail.com (C.A.); 3 Neuropsychopharmacology Unit, “Hospital 12 de Octubre” Research Institute, Avda. Córdoba, s/n, Madrid 28041, Spain; E-Mails: fjavisanzf@gmail.com (J.S.-F.); gabriel.rubio@salud.madrid.org (G.R.); 4 Department of Psychiatry, 12 de Octubre University Hospital, Avda. Córdoba, s/n, Madrid 28041, Spain; 5 Biomedical Research Center Network for Mental Health (CIBERSAM), Madrid 28029, Spain; 6 Department of Psychiatry, Complutense University, Plaza de Ramón y Cajal, s/n, Ciudad Universitaria, Madrid 28040, Spain; 7 Networks for Cooperative Research in Health (RETICS-Addictive Disorder Network), Institute of Health Carlos III (ISCIII), MICINN and FEDER, Madrid 28029, Spain

**Keywords:** clozapine, atypical antipsychotics, second-generation antipsychotics, bibliometry, schizophrenia

## Abstract

We have carried out a bibliometric study about the international scientific publications on clozapine. We have used the EMBASE and MEDLINE databases, and we applied bibliometric indicators of production, as Price’s Law on the increase of scientific literature. We also calculated the participation index (PI) of the different countries. The bibliometric data have also been correlated with some social and health data from the 12 most productive countries in biomedicine and health sciences. In addition, 5607 original documents dealing with clozapine, published between 1970 and 2013, were downloaded. Our results state non-fulfilment of Price’s Law, with scientific production on clozapine showing linear growth (*r* = 0.8691, *vs*. *r* = 0.8478 after exponential adjustment). Seven of the 12 journals with the highest numbers of publications on clozapine have an Impact Factor > 2. Among the countries generating clozapine research, the most prominent is the USA (PI = 24.32), followed by the UK (PI = 6.27) and Germany (PI = 5.40). The differences among countries on clozapine research are significantly related to economic variables linked to research. The scientific interest in clozapine remains remarkable, although after the application of bibliometric indicators of production, a saturation point is evident in the growth of scientific literature on this topic.

## 1. Introduction

Schizophrenia represents the paradigm of mental illness, due to its high prevalence as well as the severity of its symptoms and associated dysfunction. Although the pharmacological approach is only part of the management of schizophrenia, there is widespread agreement about its importance [1,2].

The origin of antipsychotic treatments is well known, from the initial serendipitous discovery of chlorpromazine in the 1950s [3,4,5]. Clozapine was also synthesized by the end of that decade [6] and was controversial from the beginning for not complying with the experimental paradigm of classical neuroleptics, what lead to coin the term “atypical” [7,8]. After its release, the evidence of its effectiveness resulted in a wide diffusion, early cut off by the appearance in 1975 in Finland of the first cases of agranulocytosis [9], which supposed its withdrawal or its restricted use in many countries. After the historical work by John M. Kane in 1988 demonstrating its efficacy in resistant schizophrenia [10], in 1990, the drug was marketed in the USA and the UK, and reintroduced in the market of countries that had previously withdrawn the product, always for the exclusive indication of resistant schizophrenia and with the requirement of harsh haematological controls [11].

Clozapine is an effective antipsychotic in schizophrenia when compared to other antipsychotic drugs [10,12,13,14,15,16,17]. In resistant schizophrenia, it is undoubtedly superior to other agents [18,19,20,21,22]. In addition, clozapine is particularly effective in certain situations, as in schizophrenic patients with suicide risk [23,24,25,26] or in reducing toxics consumption in patients with dual diagnosis [27].

Although clozapine is not free from risk, these risks have been openly oversized: after the period of initial hematologic risk of 18 weeks, and although this does not disappear [28], the risk is similar to that of taking butyrophenones and phenothiazines [29]. After the first semester, the risk of mortality by suspending the controls is the same as the risk of mortality by taking mianserin or suffering a traffic or occupational accident [30]. Overall, the risk of death due schizophrenia is far superior to death by clozapine [31]; and the use of clozapine is associated with a reduction in mortality in patients with schizophrenia [24,32,33,34].

Parallel to this evolution in the understanding and management of clozapine, the framework of prescription drugs for psychosis has evolved dramatically. Since the 1990s a number of new antipsychotics (risperidone, olanzapine, quetiapine, ziprasidone, aripiprazole, *etc*.) have appeared and progressed in the market (Table 1), designed or at least marketed in the wake of clozapine under the already confusing concept of “atypical” (atypical antipsychotic drugs, AAD). The AAD have displaced the first generation of neuroleptics almost entirely from the market, although the differences in efficacy data are not consistent [14,19,21,35,36] and radically question the homogeneity of the group [15,37,38,39,40]. Comparative studies on clozapine strongly show its superiority [16,17,19,40,41,42]. The advent of these newer agents has been accompanied by a rise in the number of scientific publications related to their pharmacology and clinical use.

**Table 1:** Clinical development of atypical antipsychotic drugs.

Drug	Company	Year	Launch
Clozapine	Wander Laboratories	1972 ^a^	Switzerland
Zotepine	Fujisawa	1982 ^b^	Japan
Amisulpride	Synthelabo	1986	Portugal
Risperidone	Johnson & Johnson	1993	UK/Canada
Sertindole	Abbott Laboratories	1996 ^c^	UK
Olanzapine	Eli Lilly	1996	USA/UK
Quetiapine	AstraZeneca	1997	USA/UK
Ziprasidone	Pfizer	2001	USA
Perospirone	Dainippon Sumitomo Pharma	2001	Japan
Aripiprazole	Otsuka/Bristol-Myers Squibb	2002	USA
Paliperidone	Janssen Pharmaceutica	2007	USA
Blonanserin	Dainippon Sumitomo Pharma	2008	Japan
Asenapine	Schering-Plough	2009	USA
Iloperidone	Novartis AG	2009	USA
Lurasidone	Dainippon Sumitomo Pharma	2011	USA

^a^ Reintroduced in 1990 in USA and UK after being withdrawn from the market in 1975; ^b^ Commercialized by Astellas in Germany in 1990; ^c^ Marketing authorization was suspended by the European Medicines Agency (EMA) in 1998 and the drug was withdrawn from the market. In 2002, based on new data, the EMA suggested that sertindole could be reintroduced for restricted use, and with extensive ECG monitoring requirement.

In this context, it is of interest to analyze the evolution of research on clozapine, within the framework of research on new antipsychotics. The use of bibliometric indicators in the study of research activity in a specific country in a particular field is based on the premise that scientific publications are the essential result of such activity [43]. Despite their methodological limitations, bibliometric studies are useful tools for assessing the social and scientific relevance of a given discipline or field [44]. Our group has studied, taking a bibliometric approach, the evolution of scientific literature in psychiatry by specific research groups, on different psychiatric disorders, and on specific therapeutic tools in the field of psychopharmacology [45,46,47,48,49,50]. Recently, we have analyzed the evolution of scientific production on AAD in Spain and in various countries of the Asia-Pacific region [51,52,53,54,55,56,57]. However, there are currently no studies that specifically address how scientific research on such a representative and unique pharmacological agent has evolved—an agent that was withdrawn from the market and whose clinical use is restricted and yet is positioned as a first-line treatment strategy in some specific cases such as resistant schizophrenia or in the case of schizophrenia with suicide risk. In the study reported here, we applied bibliometric methods to investigate trends in international publications on clozapine.

## 2. Results

**Figure 1:**
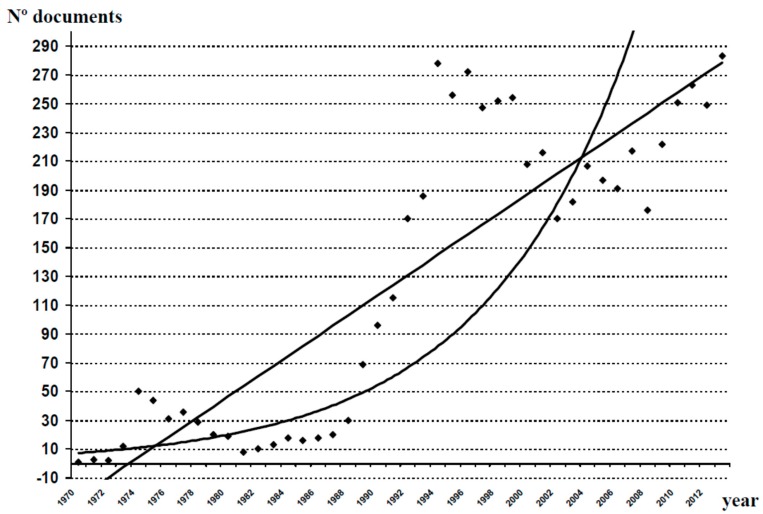
Growth of scientific production on clozapine. A linear adjustment of the data was carried out, and a fitting to an exponential curve, in order to check whether production follows Price’s law of exponential growth. Linear adjustment: *y* = 7.0338*x* − 30.828 (*r*^2^ = 0.7554). Exponential adjustment: *y* = 6.7016*e*^0.0999*x*^ (*r*^2^ = 0.7188).

**Figure 2:**
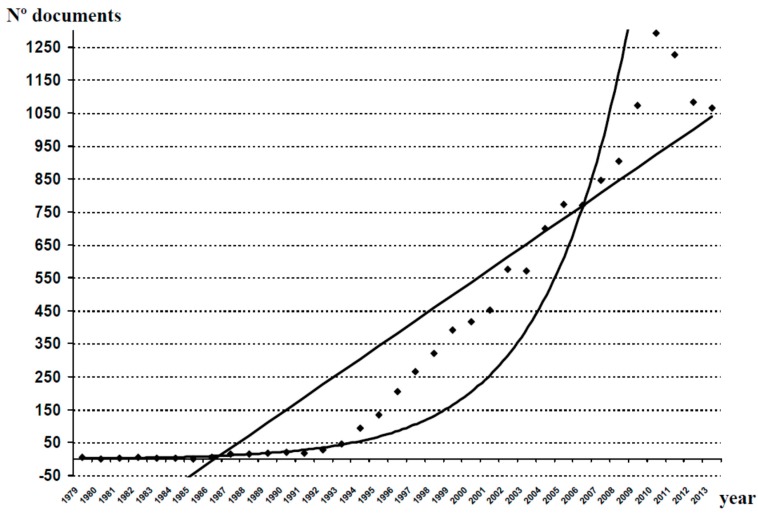
Growth of scientific production on other AADs. A linear adjustment of the data was carried out, and a fitting to an exponential curve, in order to check whether production follows Price’s Law of exponential growth. Linear adjustment: *y =* 38.718*x −* 315.56 (*r*^2^ = 0.8641). Exponential adjustment: *y =* 1.6462*e*^0.2191*x*^ (*r*^2^ = 0.9176). AADs (atypical antipsychotic drugs: Risperidone, olanzapine, ziprasidone, quetiapine, sertindole, aripiprazole, paliperidone, amisulpride, zotepine, asenapine, iloperidone, lurasidone, perospirone and blonanserin).

After the study of the databases analyzed for the period 1970–2013, we obtained 5607 original documents (articles, reviews, editorials and letters to the editor) dealing with different aspects of clozapine. As shown in Figure 1, over the last 44 years there has been a gradual increase in the number of publications generated in relation to clozapine, with some periods of decline in scientific production (1974–1985 and 1996–2008). The mathematical adjustment to an exponential curve in Figure 1, allowed us to calculate a correlation coefficient *r* = 0.8478, indicating 28.12% of variance unexplained by this fitting. In contrast, the linear adjustment of the measured values provides an *r* = 0.8691, with a portion of unexplained variance of 24.46%. With these data, we can conclude that the database analyzed was more in keeping with a linear fitting than with an exponential one, and that the postulates of Price’s Law were not fulfilled. However, when we performed this exercise in relation to the global scientific production of AADs (Figure 2), the results confirmed the fulfillment of Price’s Law (*r* = 0.9295 for a linear adjustment, 13.59% of variance unexplained, and *r* = 0.9579 for an exponential adjustment, 8.24% of variance unexplained).

**Figure 3:**
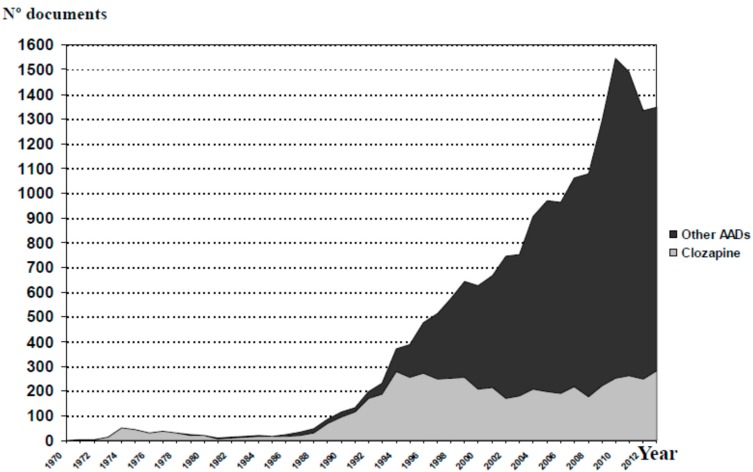
Evolution of the accumulated scientific production on atypical antipsychotic drugs.

**Figure 4:**
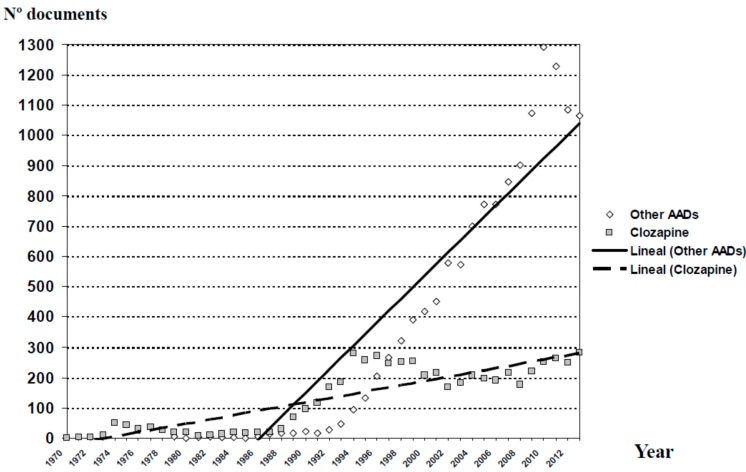
Distribution of publications on atypical antipsychotic drugs and current trend lines in relation to the scientific production from the group “clozapine” and the group “other AADs”. AADs (atypical antipsychotic drugs: Risperidone, olanzapine, ziprasidone, quetiapine, sertindole, aripiprazole, paliperidone, amisulpride, zotepine, asenapine, iloperidone, lurasidone, perospirone and blonanserin).

**Figure 5:**
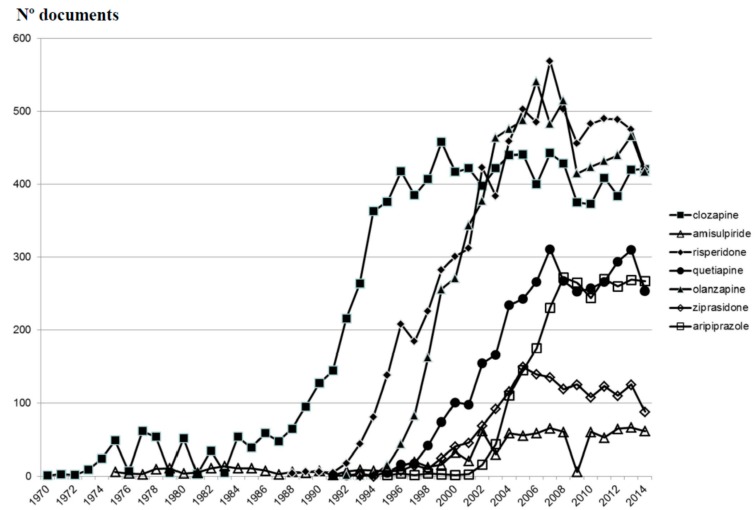
Evolution of documents on clozapine and other individual AADs.

Figure 3 shows the evolution of scientific production on AADs, including clozapine, throughout the last 44 years. The trend lines in relation to the scientific production from the group “clozapine” and the group “other AADs” are shown in Figure 4. Since 1990, the growth of publications in the group “other AADs” has been constant, whereas in the case of clozapine, we observe a flat growth since 1995. However, if we look at the individual evolution of each of these agents (Figure 5), it is evident that the scientific output of each of them is saturated from the mid-2000s. Therefore, the exponential growth of all AADs would be attributed to the overlap in time of new drug launches and the approval of new indications.

**Figure 6:**
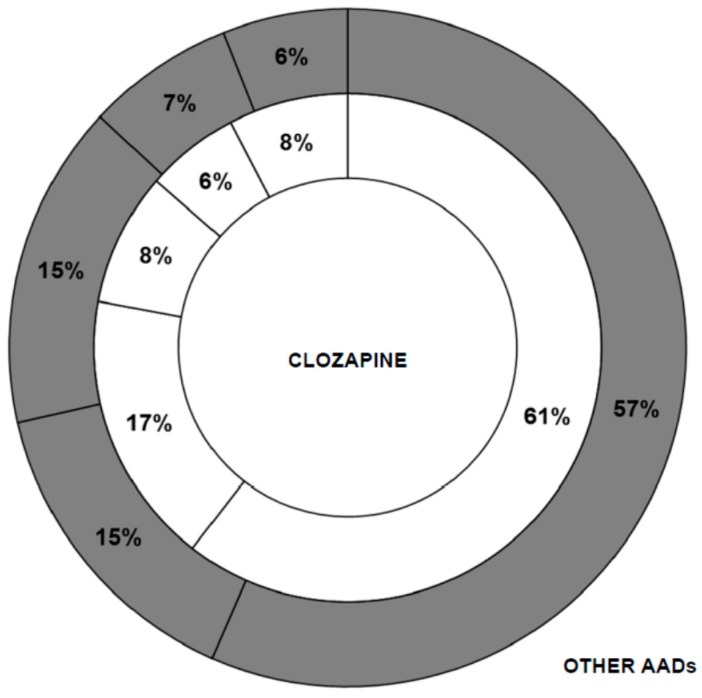
Differences in the type of publication used between the group “clozapine” and the group “other AADs”. The sections are sorted moving clockwise: Article (61%, 57%); Letter (17%, 15%); Conference document (8%, 15%); Review (6%, 7%); Others (8%, 6%).

Table 2 lists the 12 journals with the highest numbers of publications on clozapine and other AADs, and their corresponding IFs according to the Journal Citation Reports (JCR) of 2013 and the PIs of the journals on the total database in the analyzed period. It will be noted that the most extensively used journals for the diffusion of clozapine works have high IFs (seven of them has an IF greater than four). Figure 6 shows the differences in the publication types most often employed to disseminate scientific production in the field of AADs. As can be observed, there are no substantial differences between clozapine and other AADs. There are also no notable differences in the main topics of these publications (Table 3).

**Table 2:** The 12 journals with the highest numbers of publications on clozapine and other atypical antipsychotic drugs.

Clozapine			Journal	Journal	Other AADS *		
IF ^1^	PI	N° Documents			N° Documents	PI	IF ^1^
5.812	4.49	252	*Journal of Clinical Psychiatry*	*Journal of Clinical Psychopharmacology*	629	4.71	3.513
14.721	4.15	233	*American Journal of Psychiatry*	*Journal of Clinical Psychiatry*	541	4.05	5.812
3.513	3.97	223	*Journal of Clinical Psychopharmacology*	*European Neuropsychopharmacology*	454	3.40	4.595
4.061	3.24	182	*Psychopharmacology*	*American Journal of Psychiatry*	317	2.37	14.721
4.590	2.14	120	*Schizophrenia Research*	*Progress in Neuro-Psychopharmacology and Biological Psychiatry*	309	2.31	3.552
6.606	1.90	107	*British Journal of Psychiatry*	*European Psychiatry*	304	2.27	3.285
3.552	1.80	101	*Progress in Neuro-Psychopharmacology and Biological Psychiatry*	*International Clinical Psychopharmacology*	261	1.95	2.705
9.247	1.76	99	*Biological Psychiatry*	*Psychopharmacology*	246	1.84	4.061
8.678	1.62	91	*Neuropsychopharmacology*	*Schizophrenia Research*	237	1.77	4.590
3.293	1.60	90	*Australian and New Zealand Journal of Psychiatry*	*International Journal of Neuropsychopharmacology*	209	1.56	5.641
2.592	1.53	86	*European Journal of Pharmacology*	*Journal of Child and Adolescent Psychopharmacology*	209	1.56	2.773
2.109	1.39	78	*Pharmacopsychiatry*	*Neuropsychopharmacology*	207	1.55	8.678

PI (Participation Index); IF (Impact factor 2012); AADs (atypical antipsychotic drugs); ^1^ Journal Citation Report (JCR, 2013); * Risperidone, olanzapine, ziprasidone, quetiapine, sertindole, aripiprazole, paliperidone, amisulpride, zotepine, asenapine, iloperidone, lurasidone, perospirone and blonanserin.

**Table 3:** Main topics of the publications on clozapine and other atypical antipsychotic drugs *.

Clozapine (%)	Topics	Other AADs ** (%)
63.49	Drug therapy	67.94
43.32	Adverse drug reactions	49.28
26.19	Pharmacology	22.22
19.74	Endogenous compounds	19.45
14.28	Drug combination	17.75
10.98	Drug concentration	9.14
8.11	Diagnosis	9.94
7.82	Pharmacokinetics	10.11
6.70	Drug interactions	5.55
5.15	Aetiology	4.30
3.90	Drug toxicity	3.63

* Data from “Subheading” section of EMBASE; ** Risperidone, olanzapine, ziprasidone, quetiapine, sertindole, aripiprazole, paliperidone, amisulpride, zotepine, asenapine, iloperidone, lurasidone, perospirone and blonanserin.

**Figure 7:**
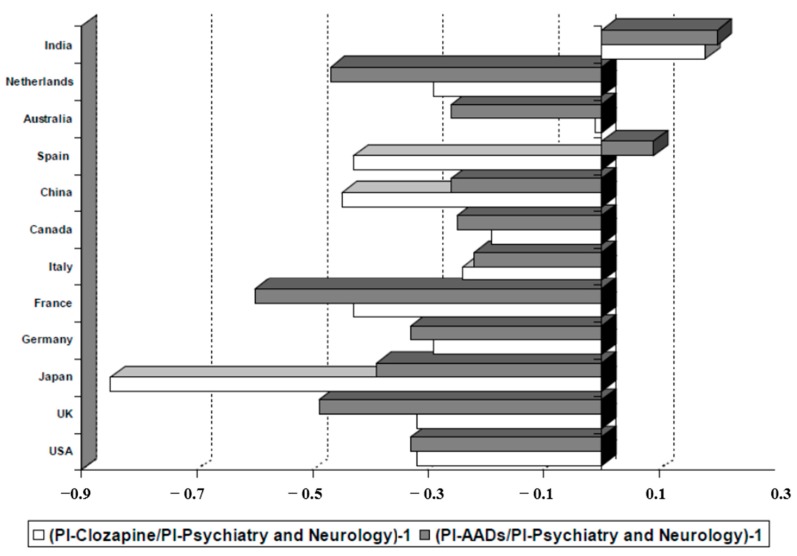
Relationship between production of scientific literature on clozapine and other atypical antipsychotic drugs (AADs) and total production in the field of psychiatry and neurology in the world’s 12 most productive countries in biomedicine and health sciences. PI (Participation Index); AADs (Atypical antipsychotic drugs: Risperidone, olanzapine, ziprasidone, quetiapine, sertindole, aripiprazole, paliperidone, amisulpride, zotepine, asenapine, iloperidone, lurasidone, perospirone and blonanserin).

As shown in Table 4, among the countries generating clozapine research, the most prominent one is the USA (PI is 24.32), followed by the UK (PI = 6.27), Germany (PI = 5.40), Canada (PI = 3.79) and Italy (PI = 3.54). However, if we consider the productivity of these countries specifically in the fields of psychiatry and neurology, only India among the 12 largest producers in biomedicine and health sciences (in the period 1970–2013) devoted a higher percentage of attention to the clozapine studies (Figure 7). In this sense, it is striking how research on clozapine in some countries, such as Japan (PI = 0.98) or Spain (PI = 1.39) is much lower than the overall research on AADs. In Australia, however, the case is the opposite, being published research on clozapine proportionally higher (PI = 2.76).

**Table 4:** Distribution of papers on clozapine and other atypical antipsychotic drugs in the world’s 12 most productive countries in biomedicine and health sciences for the period 1970–2013.

Rank	Country *	% *	Psy-Neurol ** (%)	Clozapine	Clozapine/Psy-Neurol	AADs *** (%)	AADs/Psy-Neurol
1	USA	24.91	36.16	24.32	0.14	24.08	0.34
2	UK	6.69	9.31	6.27	0.14	4.69	0.26
3	Japan	5.88	6.59	0.98	0.03	3.97	0.31
4	Germany	5.81	7.64	5.40	0.15	5.11	0.34
5	France	4.17	5.13	2.90	0.12	2.03	0.20
7	Italy	3.56	4.67	3.54	0.16	3.61	0.39
8	Canada	3.24	4.85	3.79	0.16	3.63	0.38
6	China	3.22	2.60	1.42	0.11	1.91	0.37
9	Spain	2.14	2.44	1.39	0.12	2.68	0.56
10	Australia	2.10	2.80	2.76	0.21	1.55	0.28
11	Netherlands	2.02	2.68	1.89	0.15	1.41	0.27
12	India	1.73	1.37	1.62	0.25	1.65	0.62

Psy-Neurol (area of focus in neurology and psychiatry); AADs (atypical antipsychotic drugs); * The world’s 12 most productive countries in biomedicine and health sciences for the period 1970–2013; ** Their productivity in the discipline of Psychiatry and Neurology; *** Risperidone, olanzapine, ziprasidone, quetiapine, sertindole, aripiprazole, paliperidone, amisulpride, zotepine, asenapine, iloperidone, lurasidone, perospirone and blonanserin; Total documents 1970–2013: 24,347,068; Total documents in the neurology and psychiatry area 1970–2013: 2,585,768.

**Figure 8:**
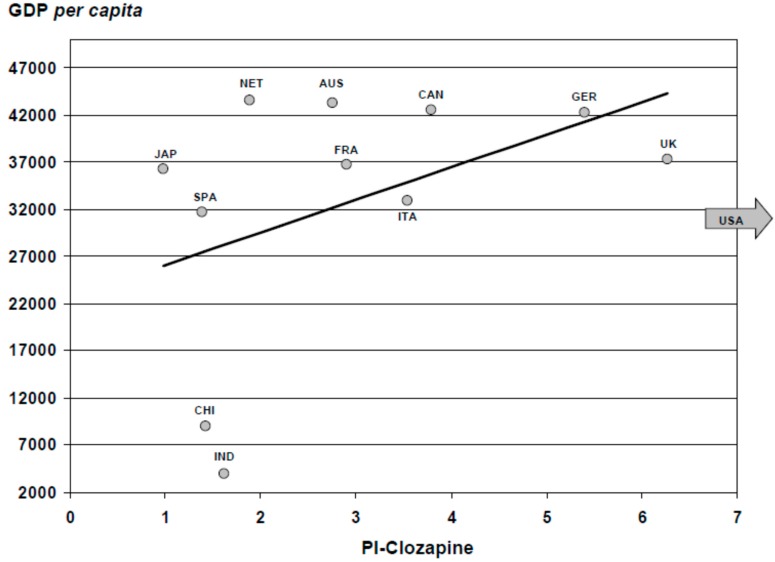
Relationship between production of scientific literature on clozapine and *per capita* gross domestic product in the world’s 12 most productive countries in biomedicine and health sciences. We have excluded the USA from the graph in order to give a clearer impression of the rest of the countries. GDP (Gross Domestic Product), PI (Participation Index). The economic data were obtained from the website of the World Health Organization (Available online: http://www.who.int/country/es/). Economic data are expressed in international dollars (data 2012). JAP: Japan; SPA: Spain; NET: Netherlands; AUS: Australia; FRA: France; CAN: Canada; GER: Germany; CHI: China; UK: United Kingdom; IND: India.

**Figure 9:**
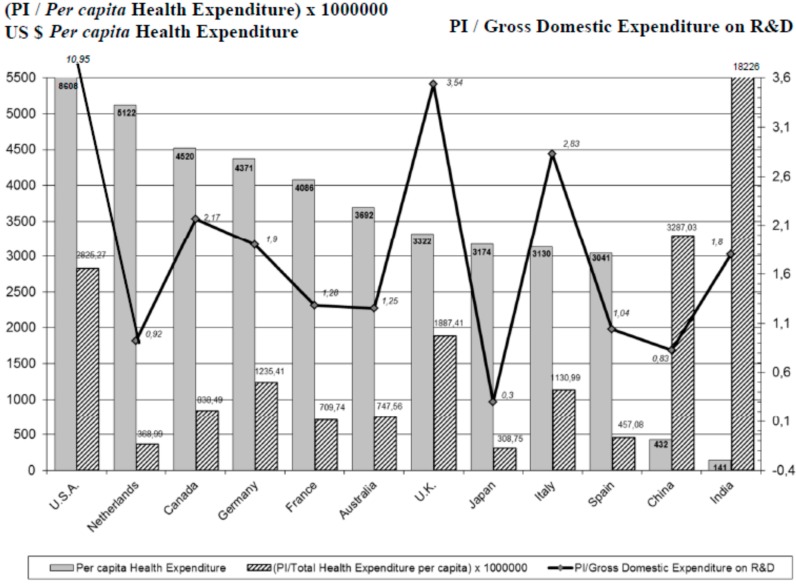
*Per capita* Health Expenditure and relationship between production of scientific literature on clozapine and *per capita* health expenditure and gross domestic expenditure on research and development, in the world’s 12 most productive countries in biomedicine and health sciences. PI (Participation Index). Total Health Expenditure *per capita* Purchasing Power Parity (PPP) Int. $ (data WHO 2011) (Available online: http://www.who.int/country/es/). Gross Domestic Expenditure on research and development (%). Data OECD 2011, except Australia and Japan (data 2010) and China (data 2009) (Available online: http://www.oecd-ilibrary.org/science-and-technology/gross-domestic-expenditure-on-r-d_2075843x-table1).

As for social-health parameters, Figure 8 shows the correlation between the PI on clozapine and the Gross Domestic Product (GDP) *per capita* of the highest scientific producers in health sciences. Analyzing the correlation between the PI and the *per capita* health expenditure of each of these countries (Figure 9), we found that the distribution was quite similar, except for India and China, although in these cases this was an artifact due to the small Indian and Chinese *per capita* health expenditure (141, and 432 Purchasing Power Parity (PPP) Int. $, respectively). However, it is striking to see what low ratios countries such as the Netherlands and Japan have.

Table 5 shows the most productive institutions in relation to the material under study. We defined the corresponding institutions solely based on the information provided in the AD field in the *EMBASE* Biomedical Answer web database. The top three ranking institutions were V.A. Medical Centers (USA), King’s College London (UK) and The Zucker Hillside Hospital (USA). The three together have generated 5.24% of the papers that make up the sample.

**Table 5:** Contribution of different institutions on clozapine.

Centre	*n*
VA Medical Center	122
King’s College London	95
The Zucker Hillside Hospital	77
University of Toronto	76
National Institute of Mental Health	59
Harvard Medical School	55
Centre for Addiction and Mental Health	49
Case Western Reserve University	48
Vanderbilt University	44
Sandoz	22

*n* (number of documents of repertoire); Data from “Affiliation” section of SCOPUS.

## 3. Discussion

Data collected on clozapine indicate that the evolution of the number of publications is better suited to a linear fit than to an exponential one, not fulfilling the principles of the Price’s law on expanding the scientific literature. For the overall analysis of the AADs, the evolution of the scientific literature is better suited to an exponential adjustment, indicating in this case, that growth has not reached the saturation point postulated by Price in his theory [58]. The first interpretation of these results indicates that, while interest in research on clozapine has stagnated, research on other AADs grows exponentially, suggesting an active scientific interest. However, this first reading could be moot, because the separate evolution of the publications of the oldest AADs (risperidone, olanzapine and ziprasidone) also reached a saturation point. We therefore consider that the exponential growth of scientific production on the overall AADs is attributable to the overlapped launch of new agents and the research generated around the latest therapeutic indications for these drugs.

Overall, the publications on clozapine do not decrease (Figure 5) but have remained on a plateau since the mid-1990s, with some decline during the last few years of the past century, which recovered strongly from 2008 on. However, we must remember that we are talking about a substance with four decades in the market, subject to a rigidly restrictive use, and with a lack of active support from the pharmaceutical industry for many years. In spite of this, the evolution of the publication of new AADs runs parallel to the succession of market launches from the beginning of the 1990s of new substances (Table 1), whose use is not restricted by any rule and which have generated plenty of publications direct or indirectly supported by the industry. Moreover, as we noted, new therapeutic indications for these agents have been recognized in the years after their commercialization. Since 2004, other AADs such as risperidone, quetiapine, ziprasidone, aripiprazole and asenapine have also been approved for treating manic episodes, and olanzapine and aripiprazole for relapse prevention in patients with bipolar disorder [59]. Quetiapine is indicated as monotherapy for the acute treatment of depressive episodes associated with bipolar disorder, and olanzapine-fluoxetine combination for treating treatment-resistant major depressive disorder. Aripiprazole was also approved in 2007 by the Food and Drug Administration (FDA) for treating treatment-resistant major depression as an add-on to an antidepressant [59]. Finally, AADs are also commonly used (and studied) for many off-label indications, such as toxic psychosis, agitation symptoms, tics, substance abuse disorders and anxiety disorders [60,61]. Therefore, we think that the steady levels of publications on clozapine over several decades suggest a special interest from researchers in this substance.

Moreover, the evolution of the consumption of AADs and the number of publications on these agents run parallel [52]. The new antipsychotics have displaced the first generation of neuroleptics almost entirely from the market, although their use is not limited to schizophrenia. For example, in Spain between 1992 and 2010, the use of antipsychotics nearly tripled at the expense of new drugs in new patients with new indications, or off-label uses [62,63]. Between July 1995 and December 2001 in Australia, the use of AADs was found to have increased from an estimated 0.27 to an estimated 3.83 DDDs/1000/day [64], and the proportion of prescribed AADs increased from 61% in 2002 to 77% in 2007 [65]. More recently, Stephenson *et al.* [66] have reported an increase of 217.7% in the dispensing of AADs in DDDs/1000/day from 2000 to 2011 (data from Drug Utilization Sub-Committee of the Australian Department of Health and Ageing). Conversely, clozapine consumption was very low; in 1999 only 25% of patients diagnosable as resistant schizophrenia in USA were treated with clozapine [67].

The disproportionate fear to clozapine side effects has led to the keeping of strict conditions of use, which, in practice, means that clozapine is not initially prescribed for many patients who might benefit from it, and the patients end up dropping off their medication due to the inconvenience of the haematological controls. Published data indicate an open under-use for the approved indication of resistant schizophrenia. If the estimated proportion of subjects with this condition ranges between 20% and 30%, clozapine prescriptions range between 1% and 2% in many countries [68]. In addition, protocols’ indications and guidelines are systematically unfulfilled, delaying the start of its use an average of five years [69], during which patients often suffer polypharmacy without evidence of efficacy [67]. Furthermore, under-dosing is frequent [70]. Finally, unlike the succession of approvals of new indications for the rest of AADs, specific indications of clozapine, such as schizophrenia with suicide risk, substance consumption or aggressiveness [71], are not officially accepted, with occasional exceptions [68]. Data from Australia differ from this trend. According to the release of the Royal Australian and New Zealand College of Psychiatrists (RANZCP) clinical practice guidelines [72], clozapine should be used early in the course, as soon as treatment resistance to at least two antipsychotic drugs has been demonstrated. However, the daily dosage of clozapine used in Australia has increased nearly 80% over the last 10 years, from 134 to 238 mg *per capita*; and 19% of schizophrenia patients in Australia are currently on clozapine therapy [73,74]. These data confirm the greater relative scientific productivity of Australia in clozapine, which are later discussed.

Similarly, some other authors employing bibliometric tools have reported that research activity in the field of schizophrenia is superior to that of other fields of psychiatry [75]. These authors also suggest that the attraction towards research on schizophrenia may have been positively affected by the clinical perception of greater severity for this illness compared to other psychiatric pathologies. Moreover, Theander and Wetterberg [76] report that the number of references on schizophrenia in MEDLINE has followed the general increase of medical publications, which accounts for a 0.42% of the total amount of medical literature in the studied period.

Regarding the main topic of the publications on clozapine, the results were surprising, since a different profile was expected for clozapine compared to other AADs, perhaps more focused on clinical issues, such as side effects, efficacy, serum levels, *etc*. However, the data provided (Table 3) indicate that the research interest is similarly oriented for both groups, despite the aforementioned heterogeneity. There are no differences in the type of work employed (articles, letters, reviews, *etc*.) to disseminate research results.

Another aspect of interest with respect to the scientific production that we have analyzed is its quality. The IF of journals used in the dissemination of works is very high, both for clozapine as well as for the rest of AADs, which leads us to highlight quality of these media. If we analyze individually the two groups regarding the 12 most used journals, we find that in the group of clozapine appear as differential sources: *British Journal of Psychiatry*, *Biological Psychiatry*, *Australian and New Zealand Journal of Psychiatry* and *European Journal of Pharmacology*. It is noteworthy how *Australian and New Zealand Journal of Psychiatry* ranks 9th, a fact which correlates with the special interest on clozapine reported in this study for Australia [56]. Furthermore, *European Journal of Pharmacology* is the only journal of general pharmacology in this ranking. The group of articles on other AADs appear as differential sources: *European Neuropsychopharmacology*, *European Psychiatry*, *International Clinical Psychopharmacology*, *International Journal of Neuropsychopharmacology* (all these are of clinical matters, where many clinical trials with AADs are published for different indications including bipolar disorder) and *Journal of Child and Adolescent Psychopharmacology*. The same subjective assessment of quality of publications could be inferred when reviewing the most productive institutions and authors on this topic.

In the analysis of the country of origin on the scientific production on clozapine and AADs (Table 4), within the group of the 12 most productive countries in biomedicine and health sciences, we note that the USA tops the ranking for production countries, generating more than a third of total number of publications in psychiatry and neurology. In this country, a fourth of the scientific production with respect to all antipsychotics (24.08%) is published, although it is a modest part of all these publications on psychiatry and neurology, and no difference between clozapine and the rest (Figure 7). The fact that this country is home to the pharmaceutical companies responsible for the development of AADs (olanzapine-Eli Lilly-, risperidone and paliperidone-Janssen Pharmaceutica-, ziprasidone-Pfizer-, and aripiprazole-Bristol-Myers Squibb-) may help to explain this high PI. Additionally noteworthy is India’s interest in the publication of AADs and clozapine, in proportion to a modest production in psychiatry and neurology. Data from Australia indicate a proportionally moderate interest on AADs, but high on clozapine, always in relation to total production in psychiatry and neurology. By contrast, Spain shows a proportional great interest in AADs but a modest one in clozapine. France, the UK and Japan have a relatively lower interest in these drugs, in the context of their overall production. The production on clozapine in Japan is very low, an expected fact since clozapine was approved in this country in 2009.

Overall, we found a higher scientific productivity of clozapine in countries with higher GDP *per capita* (Figure 8). It is also well known that the scientific productivity of a country grows proportionally to health expenditure, taking into account that the results of the investment in this field will only be evident after years, and not merely as a result of temporary economic circumstances [52]. When a particular topic is evaluated, such as productivity on clozapine, whilst keeping this trend, the results are diversified (Figure 9). Research on clozapine in relation to the Total Health Expenditure *per capita* and Gross Domestic Expenditure on research and development (%) follows the above-mentioned direct relationship (USA, UK), but with great variability (see the low proportionality in the Netherlands or Canada, and more in Italy, *etc*.), suggesting the influence of other factors that enhance research (and thus the publication) in certain countries.

We believe, based on the revised data, that the scientific interest in clozapine remains remarkable, notwithstanding the restriction factors for its use and competence with other agents. The quality of the published papers and journals that are disseminated did not differ between the two groups of AADs analyzed. The differences among countries are significantly related to economic variables associated with research, without differences between the different drugs, except for specific cases, such as Japan. It is striking to see how the proven scientific interest in clozapine, highlighted in guidelines and consensus recommendations, does not correlate with the use of this drug for the indications accepted by the scientific community.

### Limitations

The readers are warned against over-interpreting the findings of this study since it has two major limitations that are inherent to its bibliometric nature [77]. First, not all of the AAD papers were included. This bibliometric study includes papers from *EMBASE* Biomedical Answer web, and *SCOPUS*. The criteria set by the databases themselves determine the subsequent development of the studied materials [43,78]. Excluded are those papers on AADs whose authors do not put the AADs descriptors in the titles or key words of the papers, national or local journals that are not included in *MEDLINE* and *Excerpta Medica*, and contributions made at scientific conferences and meetings [46]. Secondly, the use of indicator impact factors to determine the merit or quality of scientific contributions is still debatable. In spite of the above-listed study limitations, bibliometric studies are useful in assessing the social and scientific relevance of a given discipline or field [44]. These studies constitute an effective complement to the opinions and judgments of experts in each field, providing useful and objective tools to evaluate the results of scientific activity, as well as offering a more realistic view of the whole picture and an indication of trends, and predicting how it might evolve.

## 4. Methods

### 4.1. Data Collection

The databases used in this bibliometric study were *MEDLINE* (Index Medicus, USA National Library of Medicine, Bethesda, Maryland, MD, USA) and *Excerpta Medica* (Elsevier Science Publishers, Amsterdam, The Netherlands), which are considered to be the most exhaustive databases in the biomedical field. Both participate in EMBASE Biomedical Answer web (Elsevier B.V., Amsterdam, The Netherlands). For some specific sub-analysis, *SCOPUS* database has also been used (Elsevier B.V., The Netherlands), including 55 million records, 21,915 titles, and 5000 publishers (scientific journals, books and conference proceedings).

Using remote downloading techniques, we chose documents containing, in the title (TI) section, the descriptors atypic * (atypical *) antipsychotic *, second-generation antipsychotic *, clozapine, risperidone, olanzapine, ziprasidone, quetiapine, sertindole, aripiprazole, paliperidone, amisulpride, zotepine, asenapine, iloperidone, lurasidone, perospirone and blonanserin, confining the year of publication to the period 1970–2013 (in 1970, the term “clozapine” first appeared in the title of an article). The rest of the descriptors referring to pharmacological aspects (experimental pharmacology, clinical efficacy, tolerance, safety, *etc*.) were not restricted to any field of the database and did not contribute to the inclusion criteria. For the purposes of this study, we considered all original articles, reviews, editorials and letters-to-the editor that met the inclusion criteria previously commented. All duplicated documents were eliminated: the database used allows the elimination of items that might be duplicated in both databases (MEDLINE and EMBASE).

### 4.2. Bibliometric Indicators

Among bibliometric indicators, Price’s Law is without doubt the most widely used indicator in the analysis of the productivity of a specific discipline or a particular country, reflecting a fundamental aspect of scientific production, namely exponential growth [58]. To assess whether the growth of scientific production on clozapine follows Price’s law of exponential growth, we made a linear adjustment to the data obtained, according to the equation *y* = 7.0338*x* − 30.828; and a further adjustment to an exponential curve, according to the equation *y =* 6.7016*e*^0.0999*x*^. This strategy has also been applied to the total scientific output on AADs (linear adjustment *y =* 38.718*x −* 315.56, and exponential adjustment *y =* 1.6462*e*^0.2191*x*^).

As an indicator of the publications’ repercussion, we used the impact factor (IF). This indicator, developed by the Institute for Scientific Information (Philadelphia, Pennsylvania, PA, USA), is published annually in the Journal Citation Reports (JCR) section of the Science Citation Index (SCI). The IF of a journal is calculated based on the number of times the journal is cited in the source of journals of the SCI during the previous two years and the total number of articles published by that journal during those two years. The JCR lists scientific journals by specific areas, ascribing each of them to their corresponding IF and establishing a ranking of “prestige” [79]. In this study, we used the IF data published in the JCR of 2013.

Another indicator included in the present analysis was the national participation index (PI) of the world’s 12 most productive countries in biomedicine and health sciences during the period 1970–2013 (the ratio is calculated as the number of documents on clozapine and on AADs generated by each country divided by the total number of documents generated globally). These PIs have also been compared to the global PI for biomedical and health sciences (as well as for psychiatry and neurology areas in particular). Likewise, the PIs have been correlated to some economic and health data, such as gross domestic product (GDP) *per capita*, total *per capita* expenditure on health and proportional gross domestic expenditure on research and development (R&D). The PI health data were obtained from the Organization of Economic Co-operation and Development (OECD) Health Division [80] and WHO Department of Health Statistics and Informatics [81].

## 5. Conclusions

This study offers an objective picture of the representativeness and evolution of international research on clozapine, and employs the quality and dissemination parameters most commonly used at an international level. However, research in this field will continue to grow in the coming years because the ideal antipsychotic drug has not yet been found [82], the aetiopathogeny of schizophrenia is still mostly unknown, and the clinical use of clozapine has been ever increasing.
